# Changes in the core endophytic mycobiome of carrot taproots in response to crop management and genotype

**DOI:** 10.1038/s41598-020-70683-x

**Published:** 2020-08-13

**Authors:** Sahar Abdelrazek, Sulbha Choudhari, Jyothi Thimmapuram, Philipp Simon, Micaela Colley, Tesfaye Mengiste, Lori Hoagland

**Affiliations:** 1 Department of Horticulture and Landscape Architecture, Purdue University, West Lafayette, IN USA; 2 Advanced Biomedical and Computational Sciences, Frederick National Laboratory for Cancer Research, Frederick, MD USA; 3 Bioinformatics Core, Purdue University, West Lafayette, IN USA; 4 USDA-ARS Agriculture Research Service, Madison, WI USA; 5 Organic Seed Alliance, Port Townsend, Washington, USA; 6 Department of Botany and Plant Pathology, Purdue University, West Lafayette, IN USA

**Keywords:** Microbiome, Agroecology

## Abstract

Fungal endophytes can influence production and post-harvest challenges in carrot, though the identity of these microbes as well as factors affecting their composition have not yet been determined, which prevents growers from managing these organisms to improve crop performance. Consequently, we characterized the endophytic mycobiome in the taproots of three carrot genotypes that vary in resistance to two pathogens grown in a trial comparing organic and conventional crop management using Illumina sequencing of the internal transcribed spacer (ITS) gene. A total of 1,480 individual operational taxonomic units (OTUs) were identified. Most were consistent across samples, indicating that they are part of a core mycobiome, though crop management influenced richness and diversity, likely in response to differences in soil properties. There were also differences in individual OTUs among genotypes and the nematode resistant genotype was most responsive to management system indicating that it has greater control over its endophytic mycobiome, which could potentially play a role in resistance. Members of the Ascomycota were most dominant, though the exact function of most taxa remains unclear. Future studies aimed at overcoming difficulties associated with isolating fungal endophytes are needed to identify these microbes at the species level and elucidate their specific functional roles.

## Introduction

Carrot (*Daucus carota L. subsp. sativus (*Hoffm*.) Arcang*.) is one of the most important vegetable crops in the world, providing a good source of beta-carotene, fiber, Vitamin A and other vitamins and minerals to the human diet^1,2^. Carrot taproots are often consumed raw, with per person consumption averaging 3.8 kg in 2015^3^. Organic carrot production now accounts for 14% of the U.S. market^4^, and price premiums average 15%^4^, representing an opportunity for growers to transition to organic production. However, both organic and conventional carrot growers face many challenges to produce quality crops while protecting the environment. For example, while carrots are considered a nitrogen (N) scavenging crop, a substantial amount of N fertilizers are lost to the environment^5,6^. Carrots are also subject to attack by many pests and diseases including *Alternaria dauci*^7^, and root knot nematodes^8^, as well as those that contribute to post-harvest storage losses^9^.

Endophytes, which are now commonly defined as microbes that spend at least part of their life cycle living inside plant tissues^10^, are one component of the plant microbiome that could help address these challenges. These microbes have been demonstrated to help plants acquire nutrients^11–13^, withstand abiotic stress^14,15^, and possibly even enhance the nutritional quality of crops. For example, some endophytes can produce or stimulate production of secondary metabolites^16,17^, indicating that they could play a role in the nutritional quality and organoleptic properties of plants^18^. In addition, many endophytic taxa, especially fungi, have been shown to reduce disease caused by pathogenic bacteria, fungi and nematodes^19–23^, via mechanisms that include competition, antibiosis, parasitism and induction of systemic resistance^24^. In fact, fungal endophytes could be particularly well suited to act as biocontrol agents, because they occupy the same ecological niche as invading pathogens^25^. Moreover, they would not need to compete with other soil microbes, which reduces the efficacy of many biocontrol products^26^.

While fungal endophytes clearly have potential to suppress diseases and improve performance in crops like carrot, the exact functional roles of many of these microbes remain unclear, which prevents their exploitation in agricultural systems. In addition, some fungal endophytes could negatively affect plant and possibly even human health. For example, while endophytes were originally defined as microbes that “can be isolated from surface disinfected plant surfaces” and “do not visibly harm the plant”^27^, this definition is now widely regarded as problematic because not all endophytes are culturable, and it is not easy to assess phytopathogenicity or distinguish latent pathogens from endophytes^14,15^. Moreover, some fungal endophytes can act synergistically with pathogens to facilitate infection and/or accelerate disease symptoms^19,22,28^. Antagonism appears to be the most common life history trait among fungal endophytes, though these relationships can be context dependent for reasons that are still unclear^28^. In addition, while many fungal endophytes are expected to be mutualists^29,30^, with both partners benefiting from the relationship, some appear to act as commensals gaining resources without providing any obvious benefits^31^. Finally, some endophtyic taxa with so-called ‘plant growth promoting properties’, can act as opportunistic pathogens in humans^32^. Consequently, additional studies are needed to determine how the benefits of mutualistic fungal endophytes can be leveraged, while minimizing the potentially negative effects of others.

Endophytes generally represent a subset of microbes in bulk soil, indicating that plants have some degree of control over which taxa are allowed to enter^33–35^. Nevertheless, soil is critical in shaping endophyte communities^36,37^ , since most endophytes are horizontally transmitted^19,38^. Consequently, crop management practices that alter soil microbial communities are likely to be critical in the composition and functional role of endophytes. For example, in a recent study, we demonstrated that carrot taproots grown in an organic cropping system hosted a greater abundance and diversity of culturable endophytes that could suppress *A. dauci* than carrots grown in a conventional system^39^. Another factor that can play a role in shaping plant microbiomes is plant genotype^40^. Moreover, some studies have demonstrated that microbiomes differ between genotypes that are resistant and susceptible to phytopathogens, indicating that these communities could play a role in these critical plant traits^41–43^, and this could be the case for carrot. For example, we recently conducted a greenhouse trial using field soil collected from organic and conventional management systems that were expected to be ‘disease suppressive’ and ‘disease conducive’, respectively, based on the results of our previous field trial^39^. Interestingly, only the nematode resistant genotype (E3999) had greater yield in pots containing the organic soil inoculum than those with the conventional inoculum or a sterile control^44^. Consequently, we suspect that this genotype might be able to recruit beneficial microbes when they are present in soil to aid in pathogen resistance, and/or provide other growth promoting properties such as better access to nutrients.

The development of new high-throughput sequencing technologies has made it possible to overcome limitations associated with isolating and culturing endophytic microbes and begin to investigate their potential functional role. Several studies have used these technologies to identify endophytic taxa in model crops such as Arabidopsis and Medicago^36,45^, as well as major agronomic crops such as maize^46,47^, however other important crops like carrot have been overlooked. Consequently, the objective of this study was to determine how management system and carrot genotype interact to affect the composition of fungal endophyte communities using culture-independent sequencing technologies. We predicted the following: (1) fungal endophyte communities would be more diverse in taproots grown in the organic system due to greater abundance and diversity of soil microbes; (2) carrot genotypes would host distinct communities due to differences in resistance to pathogens; and (3) the resistant genotype would be most responsive to management system, because the resistance of this genotype is due, at least in part, to its ability to recruit antagonistic fungi and/or prevent colonization of taxa that promote disease severity. To test these hypotheses, we selected three experimental genotypes that vary in resistance to root-knot nematodes and *A. dauci* (Table 1). The carrots were grown in a long-term trial comparing organic and conventional farming systems, and the composition of fungal endophyte communities in carrot taproots was identified via Illumina sequencing of internal transcribed spacer (ITS) fragments.

**Table 1:**
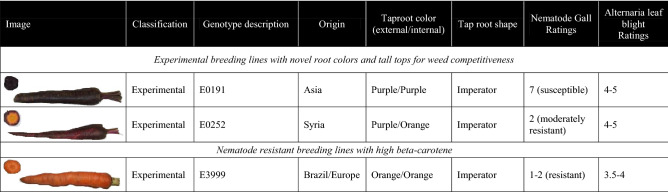
Carrot genotypes grown in conventional and organically managed systems at Purdue’s Meigs Farm during summer 2015.

## Material and methods

### Field trial

Carrot taproots were grown in a long-term crop systems trial comparing organic (ORG) and conventional (CNV) management at Purdue’s Meigs Horticultural Research Farm (lat. 40°17′21″ N. long. 86°53′02″), located approximately 10 miles south of Lafayette, IN during summer 2015^48^. Soil at this site is classified in the Drummer soil series, which typically contain approximately 3.2% organic matter and a neutral pH. The mean annual precipitation at this site is 1,008 mm, and summer temperatures range from 21.1 to 26.7 °C. The crop systems trial was established in 2011 on adjacent tracts of land with uniform topography that had previously been managed using either organic or conventional farming practices since 2001. The crop systems trial was arranged in a split-block design with three replicates for each system given constraints at the site. Within each crop system, four cash crops, carrot, tomato (*Solanum lycopersicum*), popcorn (*Zea mays everta*) and soybean (*Glycine max*), were grown annually and managed using standard practices for each system. This included application of inorganic fertilizers and synthetic pesticides in the conventional system, and inclusion of a winter cover crop and organic fertilizers in the organic system. The winter cover crop planted in the organic system consisted of a custom fall green manure mix containing winter rye (*Secale cereale L*.), hairy vetch (*Vicia villosa*), winter pea (*Pisum sativum*), annual rye (*Lolium multiflorum*), and timothy grass (*Phleum pratense*) (Cloverland Seed, Millersburg, OH). Cash crops were rotated in both crop systems annually in the following order: tomato—> carrot—> popcorn—> soybean.

In the carrot plots, fertilizers were applied to both systems to achieve a target rate of 134.5, 180 and 224 kg ha^−1^ of N, P and K respectively. In the organic plots, this consisted of Re-vita Pro Compost (Ohio Earth Foods, Hartville, OH), applied at a rate of 5,380 kg ha^−1^ to meet fertility needs, assuming 50% of the nutrients would be available for plant uptake in the year of application. In the conventional plots, diammonium phosphate (18-46-0) and potash (0-0-60) were applied to meet fertility needs. Sub-plots containing 36 carrot genotypes, which represented advanced breeding lines as well as commercial check cultivars, were randomized within each larger carrot plot, for a total of three replicates per crop system. Three of these carrot genotypes (E0191, E0252, E3999) were selected for further analysis of their endophyic mycobiome based on their country of origin, differences in top size and tap root color/shape, and resistance to pathogenic soil nematodes and *A. dauci* (Table 1). Untreated carrot seeds provided by the USDA-ARS Vegetable Crop Research Unit, Madison, WI, were planted in mid-May. Seeds were planted on raised beds that were 1.8 m apart, in 1 m rows to provide approximately 60 plants m^−1^ per sub-plot given previously determined germination rates. Seeds were sown to a depth of 1 cm. In the conventionally managed system, a pre-emergent herbicide (Prowl H2O, BASF Corporation) was applied immediately after planting. In the organically managed system, plots were hand weeded as needed. No additional pesticides were applied in either crop management system.

### Carrot screening for foliar and soil-borne pathogens

The percentage of infection by foliage pathogens in each plot was quantified using the Horsfall-Barret rating scale^49^ 60 and 110 days after seeding. In brief, the percentage of leaf area showing blight symptoms in each plot was assigned a numerical value from 1 to 12 using the arbitrary Horsfall-Barratt rating scale in which 1 = 0% infection and 12 = 100% infection. At harvest (110 days after seeding), carrots were manually harvested, and the presence of any galls or forking to indicate damage by root knot nematodes, and total number and weight of all taproots, and weight of aboveground foliar in each plot were recorded.

### Soil chemical and biological assays

Ten soil cores were randomly collected to a depth of 10 cm in each field rep just prior to carrot seeding in spring. The ten cores within each field rep were pooled and transferred to the laboratory on ice. After thoroughly mixing the cores from each replicate, a subsample of soil was air-dried before shipping to Midwest Labs (Omaha, NE) for a standard soil test according to common methods used in this region^50^. Briefly, total organic matter was determined using loss of weight on ignition; available P was extracted as Weak Bray (readily available P) and Strong Bray (potentially available P) and analyzed calorimetrically; exchangeable potassium (K), calcium (Ca), and magnesium (Mg) were extracted with neutral ammonium acetate (1 N) and quantified by inductively coupled argon plasma–mass spectrometry detection; and base saturation and cation exchange capacity [mmol ( +)·kg^−1^] were estimated from the results of exchangeable minerals^50^. Another subsample was placed in the cooler at 4 °C until being air-dried overnight to conduct assays to estimate microbial activity and active soil carbon. Microbial activity was estimated using the hydrolysis of fluorescein diacetate (FDA) in soil slurries using a method optimized for soil^51^. Active C was quantified using the permanganate oxidizable carbon (POXC) technique^52^. Finally, a subsample was lyophilized and stored at − 20, before being shipped overnight on dry ice to WARD lab (Grand Island, NE) for phospholipid fatty acid analysis (PLFA) using methods described in^53^.

### Statistical analysis of soil and plant assays

All soil chemical properties, soil microbial biomass and activity, percent infection of aboveground foliage, and number and weight of carrot roots and shoots were statistically analyzed using the general linear model procedure for ANOVA, and differences among treatment pairs were determined using the student’s t test at a p-value of 0.05, using the SAS JMP software package^54^. All data were checked for normality, homogeneity of variance and linearity prior to analysis, and were transformed when necessary.

### Fungal endophyte DNA extraction, amplification and sequencing

At harvest, two randomly selected carrot taproots representing each genotype selected for the endophytic mycobiome analysis (E0191, E0252, E3999), were collected from each of the field replicates, placed in a cooler on ice and transferred to the lab where they were stored at 4 °C until processing within 48 h. Taproots were collected from healthy plants with no signs of disease or any other plant stress. The taproots were rinsed thoroughly with tap water, then surface disinfected by soaking in 5.25% bleach for 3 min, followed by soaking in 3% peroxide solution for 3 min, and finally washing with sterilized water supplemented with 1% tween^55^. To confirm surface disinfection of the carrot taproots, 200 µl samples from the last washing solution were plated onto semi-selective media for heterotrophic bacteria (Tryptic Soy Agar), oligotrophic bacteria (R2A), and total fungi (1/5th PDA media)^56,57^, each with two replicates. The carrot cores were also rolled over the surface of each semi-selective media. The petri plates were incubated at 27 °C or 25 °C and counted after 48 or 72 h, for bacterial and fungal enumeration respectively. Five (15 mm) carrot cylinders were collected from each taproot using a sterilized core borer, and the five cores from each field replicate were pooled for analysis. Carrot core samples were lyophilized (LABCONCO, Kansas City, U.S.A) and stored at − 80 °C until DNA extraction.

Endophyte community DNA was extracted in duplicate from each lyophilized carrot root sample using Qiagen DNeasy Plant Mini Kits (Qiagen, U.S.A) following the manufacturer’s protocol and diluted using 100 μl of elution buffer. The two lab replicates were pooled, and DNA was quantified using a Qubit Fluorometer 2.0 and dsDNA HS Assay Kit (Thermo Fisher Scientific, U.S.A.) and normalized to 1 ng/μl prior to ITS amplification. Fungal endophyte community ITS library construction was carried out in two steps. First, the ITS1 region was amplified using the universal primers ITS1F forward primer 5 ′CTTGGTCATTTAGAGGAAGTAA-3′^58^ and ITS2 reverse primer 5′-GCTGCGTTCTTCATCGATGC-3′^59^ modified to contain an adapter region for sequencing on the Illumina MiSeq platform, in triplicate reactions for each sample. Each 25-μl PCR reaction mixture contained 3 μl of DNA template, 0.5 μl (100 mM) of each primer, 12.5 μl GoTaq colorless Master Mix (Promega, Wisconsin, U.S.A) and 8.5 μl of nuclease free water (Promega, Wisconsin, U.S.A.). Each PCR reaction was performed using a Bio-Rad T100 Thermal Cycler (BioRad, California, U.S.A) with the following conditions: initial denaturing using 1 cycle at 95 °C for 2 min, 40 cycles of the following (denaturing step 95 °C for 30 s, annealing step 55 °C for 30 s, and extension step 72 °C for 1 min), and a final extension step of 72 °C for 10 min. Detection of PCR-amplified products was performed with electrophoresis on a 0.7% (wt. /vol.) agarose gel stained with Bullseye DNA Safe Stain (MIDSCI, U.S.A.). A 100 bp ladder (New England bio lab, U.S.A) was also run in parallel to approximate PCR product band sizing. Presence of DNA bands stained with DNA Safe Stain (MIDSCI, U.S.A.) were visualized after exposure of the gel to ultraviolet (UV) light. PCR replicate products of the same samples were pooled and cleaned using Ultraclean PCR Clean-Up Kits (MO BIO, U.S.A) following the manufacturer’s protocol. Cleaned PCR products were subjected to a second PCR reaction, with specific tag encoded primers for each sample. The same thermocycling conditions described above were used, with the exception of 5 amplification cycles instead of 35. Again, all PCR products were confirmed by electrophoresis as described above. Final PCR product concentration was quantified and adjusted using the Qubit Fluorometer 2.0 as described above. Samples were submitted in equimolar concentrations (20 ng) to the Purdue Genomics Facility for sequencing of ITS libraries. A TruSeq DNA LT Sample Prep Kit (Illumina, San Diego, CA) was used to construct paired-end (2 × 250 bp) sequencing libraries. MiSeq Reagent Kit v2 (Illumina, San Diego, CA) was used to perform amplicon sequencing on a MiSeq Desktop Sequencer (San Diego, CA).

After demultiplexing, the reads were quality-filtered, converted to FASTA format using FASTX-toolkit (Version 0.0.14), and concatenated into a single file for use as an input into QIIME (Version 1.9.1)^60^. The reads with Phred quality score of Q30 were retained for further analysis. Operational taxonomic unit (OTU) picking, taxonomic assignment, and construction of phylogenetic trees were carried out using QIIME’s open-reference OTU picking module using the UCLUST method^61^. Reads were clustered against a reference fungal database (UNITE 97, 12_11 version) at 97% identity, and reads that failed to hit the reference were subsequently clustered de novo into operational taxonomic units (OTUs). All the samples were taken into account without any subsampling. The suppress_align_and_tree was passed as a parameter because the trees generated from ITS sequences are generally not phylogenetically informative. Only OTUs of fungal origin were considered for further analysis. The QIIME module identify_chimeric_seqs.py that employs the Chimera Slayer algorithm^62^ was used to screen for chimeric sequences. To report the number of sequences per sample, the QIIME module biom summarize-table was used. To estimate the alpha diversity within the taproots of three carrot genotypes grown under organic and conventional management , the alpha diversity script based on Faith’s phylogenetic diversity index^63^ was used. The two-sample *t*-test was used to determine the diversity between genotypes under different soil management. The diversity in the samples was calculated using three different diversity indices: Observed OTUs, Chao-1 Estimator^64^, and PD_whole_tree^65^ and sequencing depth was assessed using rarefaction curves.

QIIME’s filter scripts were used to retain OTUs where 25% of the samples in groups being compared have OTUs. Beta diversity estimates were calculated within QIIME using Bray–Curtis distances matrices and results were used to produce principle coordinate analysis (PCoA) plots to visualize differences^66^. Community differences within all samples of a group as well as between different groups were further assessed using t-tests, while community differences between groups were assessed using QIIME’s compare_categories.py script and ADONIS methods^67^.

In order to quantify differential abundance for specific OTUs between groups among the different comparisons, the phyloseq software package, implemented in Bioconductor, was used to provide a platform for statistical analysis and figure generation in R For each comparison, p-values were adjusted for the false-discovery rate (FDR) and OTUs with adjusted p-values below 0.2 were considered significant and were used to generate ggplot2 summary plots. Finally, to determine which fungal OTUs best characterized taproot endophyte communities as a function of management system, carrot genotype, and the interaction of these two factors, we used an indicator species analysis in the labdsv package in R^68^. Indicator species values are based on how specific and widespread an OTU is within a particular subgroup and are independent of the relative abundance of other fungal taxa in carrot taproots^69^.

## Results

### Impact of management system on soil properties, disease severity and yield

**Figure 1:**
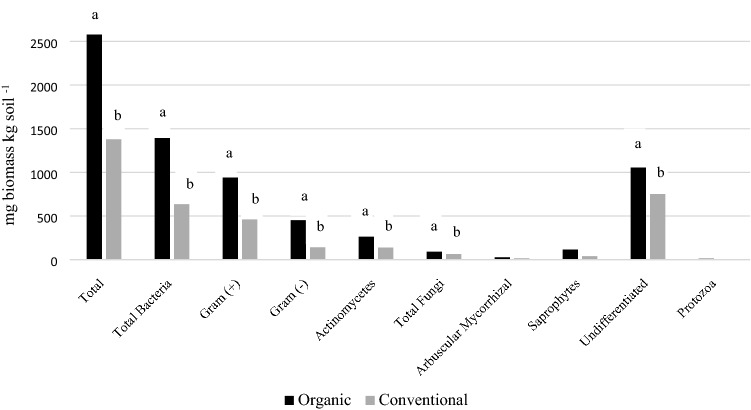
Microbial biomass estimated using soil phospholipid fatty acid analysis (PLFA) in soil collected from carrot plots grown using organic and conventional management at Purdue’s Meigs Farm during summer 2015. ^z^Different letters within a column represent significant difference as determined by Tukey’s honestly significant difference test (P < 0.05).

Soil pH, total and active organic matter, calcium and percent calcium on cation exchange sites (CEC) were significantly greater in the organic system, while percent hydrogen on CEC sites was significantly greater in the conventional system (Table 2). Many components of the microbial biomass including total microbes, total bacteria, gram positive and negative bacteria, actinomycetes, and total fungi were greater in the organic system (Fig. 1). The severity of leaf blight caused by foliar pathogens was high during summer 2015 in the carrot plots as all carrot genotypes had between 75–90% infections just prior to harvest (119 days after seeding), but there were no significant differences between carrot genotypes or management system (Table 3). Pathogens isolated from carrot foliage in both of these cropping systems have previously been identified as *A. dauci*, *Cercospora carotae* and *Xanthomonas campestris*^70^ (duToit, personal communication). There were no visible symptoms of nematode infection in any of the three carrot genotypes evaluated in this study regardless of the susceptibility or resistance to nematodes (Table 3). Total shoot and root weight in genotype E0252 was greater in the conventional than organic system, and the shoot and root weight of E0252 was greater than E3999 (Table 3).

**Table 2:** Soil chemical properties, active organic matter and microbial activity in carrot field managed using organic and conventional farm practices just prior to planting in summer 2015 at Purdue’s Meigs Horticulture Research Farm.

Crop system	%OM	P-weak bray	P-strong bray	K	Mg	Ca	pH	CEC	%K	%Mg	%Ca	%H	POXC	FDA
		ppm							Percent base saturation				mg POXC/kg soil	ug FDA/g soil/h
Organic	3.1 a^z^	34.3	67.7	230.0	426.3	2,790 a	6.7 a	19.2	3.1	18.3	72.8 a	4.5 b	395.2 a	0.162
Conventional	2.2 b	70.7	81	256.3	335.7	1991 b	6.0 b	16.0	4.1	17.5	62.6 b	15.7 a	294.9 b	0.122

^z^Different letters within a column represent significant difference as determined by Tukey’s honestly significant difference test (P < 0.05).

**Table 3:** Carrot biomass, percentage of damage by foliar pathogens and nematode diseases severity in organic and conventional field trials during summer 2015.

Management system	Carrot genotype	% Damage by foliar pathogens		# of plants	Nematode rating	Plant biomass at harvest (g)			
		60 day	119 day			Shoots		Roots	
Organic	E0191	0.0	71.7	8.3	0.0	0.19	A	0.57	A
Conventional	E0191	3.3	83.3	5.3	0.0	0.23		0.59	
Organic	E0252	5.0	90.0	3.7	0.0	0.05 b	A	0.14 b	AB
Conventional	E0252	0.0	66.7	9.7	0.0	0.22 a		0.64 a	
Organic	E3999	23.3	91.7	5.7	0.0	0.02	B	0.12	B
Conventional	E3999	25.0	83.3	7.3	0.0	0.04		0.27	

^z^Different letters within a column represent significant difference as determined by Tukey’s honestly significant difference test (P < 0.05).

### Abundance and quality of fungal endophyte sequences

After quality filtering, adapter trimming, and merging of Illumina reads, approximately 3,793,627 high-quality sequences were obtained and used as input for analysis and comparison of fungal endophyte communities. Sequences clustered into 1,480 different fungal operational taxonomic units (OTUs) when grouped at the 97% genetic similarly level (Table S1). Rarefaction curves (Fig. S1) indicated that only 38.5% of fungal endophyte diversity present in carrot taproots was recovered by this surveying effort.

### Assignment of OTUs to fungal taxa

**Figure 2:**
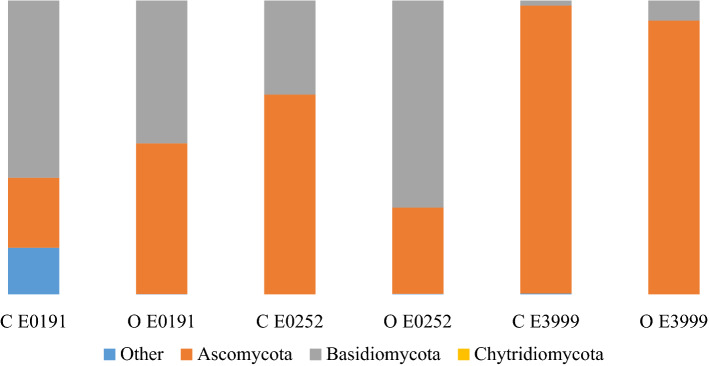
Relative abundance of fungal endophytes by phyla in the taproots of three carrot genotypes grown under conventional (C) and organic (O) management.

**Figure 3:**
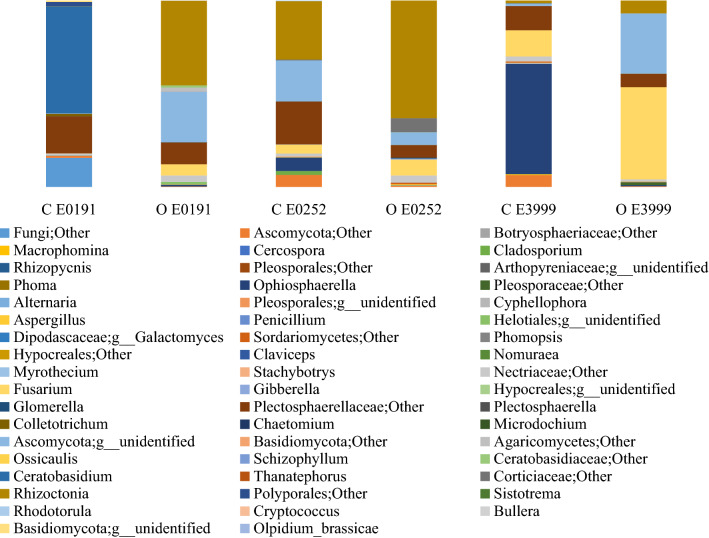
Relative abundance of fungal endophytes by genera in the taproots of three carrot genotypes grown under conventional (C) and organic (O) management.

Carrot taproots were dominated by microbes in the Ascomycota phyla (73.9%) (Fig. 2). Other abundant phyla belonged to the Basidiomycota (24.8%) and Chytridiomycota (< 1%) (Fig. 2). At the level of genera, *Rhizoctonia* and Fusarium were predominant, representing 19% and 13% of all endophytes identified (Fig. 3). Other taxa observed across all samples included *Ophiosphaerella* (5.4%), *Ceratobasidium* (3.6%), *Colletotrichum* and *Gibberella* (each at 0.4%), *Cladosporium* (0.3%), *Aspergillus* (0.2%), and *Cyphellophora*, *Thanatephorus*, *Alternaria* and *Plectosphaerella* (all at 0.1%). Finally, *Cercospora*, *Rhizopycnis* and *Phoma* were among twenty other genera observed with less than 0.1% relative abundance.

### Effect of crop management system on fungal endophytes

**Figure 4:**
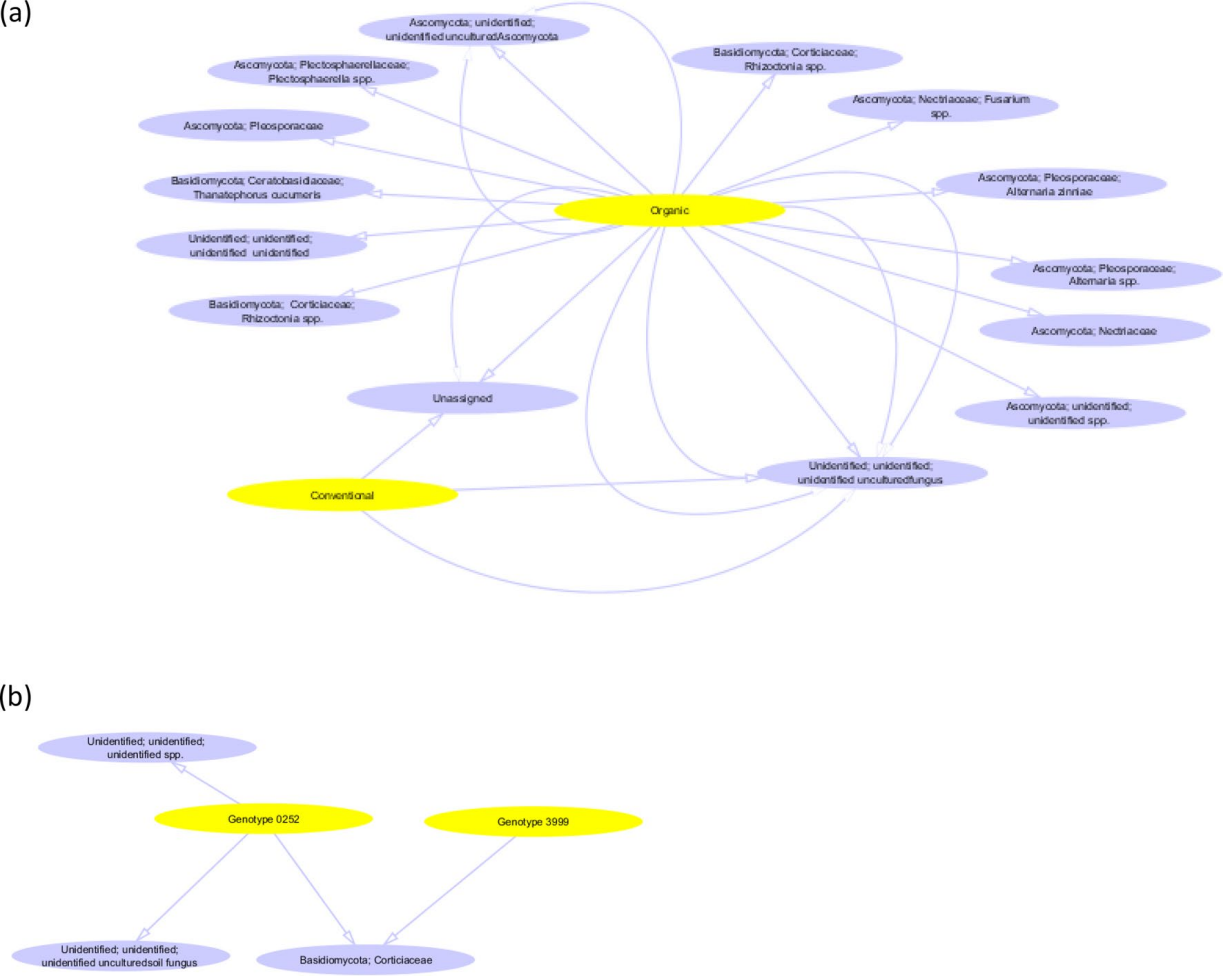
Indicator species analysis identifying individual fungal OTUs in carrot taproots; (**a**) Fungal OTUs unique to management system, (**b**) Fungal OTUs unique to carrot genotype.

Fungal endophyte richness (Table 4a) and beta diversity (Table 4b) were significantly greater in the organic management systems, but alpha diversity was not (Table 4a). Of the 1,480 individual fungal endophyte OTUs identified, 98.3% were not significantly different in relative abundance or frequency with respect to management system (Fig. 4a & Table S2). However, individual OTUs representing *Ascomycota*, *Basidiomycota* and *Chytridiomycota* phyla, which comprised 1.6% of all fungal taxa observed in the study, were specifically associated with one management practice (Fig. 4a and Table S2). Of these, 87.5% were significantly associated with organic management, while only one unidentified, unassigned and uncultured fungal genus was significantly associated with conventional management. At the level of genera, the indicator species analysis indicated that genera belonging to *Alternaria, Fusarium Plectosphaerella, Rhizoctonia* and *Thanatabasidium* were uniquely correlated with organic management, while only one unidentified and one unassigned species was uniquely correlated with conventional (Fig. 4a).

**Table 4:** (a) Influence of crop management systems, carrot genotype and their interactions on fungal endophyte richness and alpha diversity within the taproots of three carrot genotypes grown under organic and conventional management. (b) Influence of crop management systems, carrot genotype and their interactions on fungal endophyte beta diversity within the taproots of three carrot genotypes grown under organic and conventional management.

(a) Comparison	Richness	Diversity
	p-value	p-value
Management system	0.019	0.354
Carrot genotype	0.778	0.205
Management system + carrot genotype	0.284	0.524
Management system + E0191	0.275	0.513
Management system + E0252	0.513	0.827
Management system + E3999	0.050	0.275

(b) Comparison	Bray–Curtis	
	p-value	
Management system	0.030	
E0191 vs. E0252	0.743	
E0191 vs. E3999	0.720	
E0252 vs. E3999	0.667	
Conventional E0191 vs. conventional E0252	0.801	
Conventional E0191 vs. conventional E0252	0.800	
Conventional E0252 vs. Conventional E3999	0.901	
Organic E0191 vs. Organic E0252	0.801	
Organic E0191 vs. organic E3999	0.801	
Organic E0252 vs. organic E3999	0.900	
Conventional E0191 vs. organic E0191	0.201	
Conventional E0252 vs. organic E0252	0.900	
Conventional E3999 vs. organic E3999	0.101

### Effect of carrot genotype on fungal endophytes

Carrot genotype did not affect fungal richness, alpha (Table 4a), or beta diversity (Table 4b) when genotypes were compared across management systems. However, several individual fungal genera including unidentified and non-assigned genera, *Cladosporium, Thanatephorus, Rhizoctonia, Ceratobasidium, Aspergillus, Cyphellophora* and *Ophiosphaerella* differed among genotypes (Fig. S2). Specifically, there was a greater abundance of *Cladosporium, Thanatephorus, Rhizoctonia, Ceratobasidium* and *Aspergillus* in E0191 when compared with E3999, whereas the opposite occurred with *Cyphellophora* and *Ophiosphaerella* genera. E0191 also had a greater abundance of *Aspergillus* and *Ceratobasidium* than E0252. In contrast, only a few unidentified and non-assigned genera were more abundant in E0252 than E3999 (Fig. S2). The indicator species analysis indicated that only three out of the 1,480 fungal taxa were correlated with an individual carrot genotype (Fig. 4b and Table S3). This included one uncultured fungus and one unidentified fungus that were uniquely associated with E0252, and one fungal taxon related to the Corticiaceae family, that was correlated with E0252 and E3999.

### Interactions between carrot genotype and management system on fungal endophytes

Only genotype E3999 had differences in taxonomic richness of fungal endophytes (P < 0.05) (Table 4a), and marginal differences in beta diversity (P < 0.10) (Table 4b) when grown in the two contrasting production systems. There were differences in the abundance of individual fungal endophyte OTUs in E0191 and E3999 grown in the two management systems (Fig. 3; Fig. S2). Specifically, within E0191, the relative abundance of *Aspergillus, Ophiosphaerella, Rhizoctonia, Thanatephorus* and *Fusarium* were significantly greater in carrots grown in the organic system whereas the opposite was found with *Colletotrichum* and *Ceratobasidium*. Within E3999, seven fungi including an uncultured Ascomycota, an uncultured fungus, and some non-assigned taxa were significantly greater when grown under organic compared to conventional management, while the opposite was found for one uncultured fungus and one non-assigned fungus. In contrast, no differences in individual genera were detected in E0252 when grown in the organic compared to conventional management system (Fig. S3).

When comparing differences among genotypes within each individual management system, there were differences in the abundance of some genera (Fig. S2). Specifically, under conventional management, a greater relative abundance of *Ophiosphaerella* and *Cladosporium* genera were present in E0252 than E0191, whereas the opposite was found for *Ceratobasidium. Ophiosphaerella* was more abundant in E3999 than either E0191 or E0252, along with a few other unidentified and non-assigned genera (Fig. S2). Under organic management, *Aspergillus* and *Rhizoctonia* were more abundant in E0191 than E3999, one unidentified genus had greater abundance in E0191 than E0252, and one non-assigned genus had greater abundance in E0252 than E3999 (Fig. S2).

## Discussion

Results of this study confirm earlier reports^9,39^ indicating that carrot taproots are colonized by a diverse assortment of fungal endophytes, with the majority belonging to the Ascomycota phyla (Fig. 2). Members of the Ascomycota are common as endophytes in the roots of a wide variety of plant species in ecosystems ranging from the arctic tundra, to tropical forests and croplands^9,71–75^. In a recent review of all eukaryotic ITS sequences available, the Ascomycota represented 30% of all fungal endophytes in plant roots identified to date^14^. It is unclear why these fungi are so predominant in plant roots and especially in carrot, though it could have something to do with their close relationship with many pathogens in the same phyla^14,76^. To enter and survive inside plants as endophytes or pathogens, microbes must possess plant-degrading enzymes, and/or be able to silence plant defense pathways^36,77^. Over the course of evolution, there are many examples of transitions between endophytic and pathogenic life history traits among the Ascomycota^78^. Consequently, endophytism could remain a viable life history strategy if members of the phyla act as ecological opportunists to form pathogenic relationships when environmental conditions make this strategy better for their long-term survival^78^. This could also help explain why many members of this phyla act as pathogens in one plant species and endophytes in another^14^, especially if they are obligate microbes that cannot survive or reproduce in soil.

Other prominent fungal phyla in carrot taproots included members of the Basidiomycota (Fig. 2). Fungal taxa within the Basidiomycota also include mutualists and commensals as well as pathogens^14,19^, so their predominance in carrot taproots could also be related to their ability to transition between endophytism and parasitism. Surprisingly, we did not observe any fungi from the Glomeromycota, despite the fact that they are generally the most abundant fungal endophytic phylum in plant root surveys^14^. Members of the Glomeromycota form arbuscular mycorrhizas, which are well known for their potential to help plants, including carrot, obtain nutrients and withstand biotic and abiotic stress^79,80^. This could be due to the fact that the primer sets we used are not ideal for amplifying this fungal phylum^58,81,82^, as well as that our samples were from carrot taproots rather than fine roots where mycorrhizal fungi are generally more common^9,83^. Unfortunately, many of the OTUs obtained in this study were characterized as either unidentified or unassigned, highlighting the challenges associated with the lack of informative sequences in existing fungal databases^82,84^.

As 98.3% of the fungal taxa identified in this study did not differ between the two management systems (Table S2; Fig. S2), these taxa likely represent a ‘core mycobiome’ in carrot taproots. A plants core microbiome represents a set of microbial taxa that are systematically associated with a given host plant^85^. In many cases, these core microbiomes appear to remain relatively stable over the course of evolution and domestication^12,36,86^, at least with respect to broad taxonomic groups^14^. Nevertheless, soil can influence the composition of endophytes^34,35,37^, especially at finer taxonomic scales. Results of our study provide further support for this phenomenon, by demonstrating that the diversity of fungal endophytes in carrot taproots is dependent on crop management systems that differ in soil chemical and biological properties (Tables 2 and Fig. 1). Other studies have also provided evidence that differences in soil characteristics induced by management practices are a strong driver of endophyte composition^55,87,88^. In particular, management practices commonly used in organic and conventional farming systems are well known for their potential to alter many soil properties^89^. For example, organic farmers commonly plant cover crops and apply organic fertility amendments, which increase soil organic matter and serve as the primary food and energy source for soil microbes^89–91^. Consequently, as the soil in the organic management system in this study had more active organic matter and a greater abundance of several types of soil microbial biomass including fungi (Table 2 and Fig. 1), it is not surprising that endophytes were more diverse in taproots grown in this system (Fig. S2). Others have suggested that differences in fungicide applications between organic and conventional systems could also affect endophyte composition^87,92^, however, this is not likely to be the case in this study, as we did not apply any fungicides in either system.

While clarifying the specific functional roles of fungal endophytes in carrot taproots will require additional studies using taxa that have been isolated and cultured, it is possible to begin to speculate about their potential functional roles given results of this sequencing effort. Several individual OTUs were uniquely associated with the organic system (Fig. 4a). While fungi associated with these genera have been implicated as pathogens in some crops, they have also been isolated from healthy plant tissues in other species and demonstrated to provide benefits^14,19^, indicating that they might not necessarily act as pathogens in carrot. For example, *Plectosporella* species have been isolated from healthy soybean^93^, vegetable^94^, and quinoa^95^ roots. While some *Plectosporella* isolates caused disease symptoms when inoculated onto lettuce, others increased plant growth^94^. In another study, an endophytic *Plectosporella* isolated from carrot taproots failed to produce any disease symptoms when re-inoculated onto new carrot plants^96^, indicating that these taxa may not act as pathogens in carrot and instead could provide benefits.

It is possible that some of the taxa that were more abundant in the organic taproots such as *Rhizoctonia,* are latent pathogens and/or could contribute to diseases caused by other pathogens. However, we do not expect that this was the case here. In our previous study isolating culturable endophytes from carrot taproots grown in the same organic and conventional fields, foliar disease incidence was lower in the organic system in two of the genotypes evaluated in this study (E0191 and E0252)^39^. Moreover, soils in the organic system had greater microbial biomass and activity, and endophytic isolates collected from roots grown in the organic system had greater antagonistic activity against *A. dauci*. Consequently, because several soil biological properties were also greater in the organic system in this study (Table 2 and Fig. 1), we expect that microbes in these soils could have been more suppressive against pathogens, and/or had other plant growth promoting properties. Other studies have demonstrated that soils in organic farming systems can be more disease suppressive than their conventional counterparts^97,98^, and microbes isolated from the rhizosphere of plants grown in organic systems have greater potential to suppress diseases^99^. Endophytesisolated from vegetables grown under organic management have also been shown to be more abundant and diverse, and have greater growth promoting properties than those grown in conventional systems^88^. Finally, the fungal endophytes in this study were collected from healthy plants in a year where foliar disease pressure was very high (Table 3), thus we expect that they were not pathogens and instead could have played a role in helping carrots resist diseases, though future studies are needed to verify this hypothesis.

Like pathogens, plants are able to sense and respond to the presence of endophytic microbes, acting as ‘gate keepers’, to exclude or permit different taxa from entering and persisting in plant roots^77,100^. Consequently, it is not surprising that plant genotype can also play a smaller, yet significant role in shaping plant microbiomes^40,101^, and carrot is not an exception (Figs. S2, S3). Over the course of evolution and breeding, plants experience different selection pressures which could influence whether the presence of endophytic taxa are maintained^15^. For example, fungal diversity in plant genotypes selected in modern agricultural systems has been reported to be lower than in wild ancestors, in a phenomenon referred to as “domestication syndrome”^102^. In contrast, it is also possible that selection for traits such as disease resistance could have inadvertently selected for microbes that aid in plant resistance. For example, targeted breeding efforts have resulted in the development of carrot genotypes that are highly resistant to root knot nematodes^103^. Mechanisms appear to include: (1) differences in chemical cues attracting nematodes to roots, and the ability of nematodes to (2) penetrate the epidermis, (3) migrate through the root surface to establish a feeding site in the vascular parenchyma, (4) develop root galls, and (5) reproduce^103^. While some of this resistance is likely regulated by specific R genes, such as those that mediate a hypersensitive response at the root surface when pathogens attempt to enter host tissue, other components could be mediated, at least in part, by endophytes. For example, while host genes for resistance in *Populus* represent the strongest and first line of defense against pests, antagonism by fungal endophytes represents an important second line of defense^37^. Interestingly, resistance to root knot nematodes in carrot appears to be mediated post-infection^103^, thus it is plausible that fungal endophytes could play a role in preventing nematodes from migrating, forming galls and/or reproducing. The two genotypes that differed most in this study with respect to differences among individual OTUs were E0191 and E3999 (Fig. S2, 3), which are susceptible and resistant, respectively, to pathogenic nematodes. Previous studies have demonstrated that fungal endophytes can suppress disease caused by pathogen nematodes^23^, therefore it is possible that differences in these endophyte communities could play a role in preventing, or facilitating, the infection and severity of pathogenic nematodes in these genotypes. However, they also differ in taproot color (Table 1), and E0191 had significantly greater yield than E3999 (Table 3), so it is also possible that these factors could have contributed to the differences observed in this trial.

Several individual OTUs were significantly greater in the susceptible (E0191) than resistant (E3999) genotype (Fig. S2). Isolates of both *Rhizoctonia* and *Ceratobasidium* have been shown to cause disease or disease like symptoms in carrots^104,105^, though *Ceratobasidium* has also been reported to act as a mycorrhiza in orchids^106^, and suppress diseases in rice^107^ and cacoa^108,109^. *Cladosporium* is a pathogen in spinach^110^, though these taxa can also enhance plant growth in soybean^111^. Members of the *Aspergillus* genus have been demonstrated to increase growth and reduce soft rot in carrot plants^112^. *Aspergillus* taxa can also produce bioactive products active against many phyto as well as human pathogens^113,114^, indicating that they could enhance plant as well as human health. However, *Aspergillus* spp. can also cause human health problems and contribute to reductions in post-harvest quality in carrots^115^, so isolates of this particular genus would need to be carefully tested before they could be considered for use as inoculants to improve carrot performance^32^. Three individual OTUs were enriched in E3999 relative to E0191 (Fig. S2). *Ophiosphaerella* spp. are well known for their potential to act as a pathogen in bermudagrass^116^, and *Cyphellophora* endophytes are suspected to play a role in facilitating apple diseases^117^. However, *Ophiosphaerella* spp. can solubilize calcium, aluminum and iron phosphates^118^, indicating that they could play important roles in plant nutrition. Endophytic isolates of *Cyphellophora* were isolated from plants grown on heavily contaminated mine tailings, indicating that they could play a role in helping plants tolerate abiotic stress^100^. Members of the *Corticaceae* are often reported as endophytes in woody plants such as Populus^101^, though their potential functional role remains unclear. Clearly, there is still much work to do to decipher the actual roles of these fungal taxa in carrot taproots, though now that these taxa have been identified using NGS sequencing, it will be possible to design future studies to isolate these taxa and elucidate their specific role.

We predicted that E3999 would be most responsive to differences in soil microbial communities induced by the management systems evaluated in this trial, because of its disease resistance and the fact that we previously noted increased growth in this genotype in the presence of soil inoculum from the organic system in a controlled trial^44^. Interestingly, the results of our sequencing efforts support this hypothesis, as E3999 was the only genotype that differed in richness between the two management systems (Table 4a), and there were marginal differences in beta diversity (Table 4b). As described above, organic farming systems can host microbes that promote plant growth and have greater disease suppressive activity than their conventional counterparts^39,88,97–99^. Consequently, we suspect that there could have been greater populations of fungi with suppressive and/or plant growth promoting activity available in the organic system that could have been recruited by E3999 to help this genotype fight pathogens or improve its growth. Alternatively, it is possible that at least part of the resistant activity of this genotype is due to its ability to restrict entry by endophytic microbes that do not directly cause disease but promote the colonization, survival or virulence of pathogens as part of a pathobiome^119^. Future studies testing these and other hypotheses are needed to determine the extent to which endophytes can mediate disease dynamics.

We also observed differences in individual OTUs in E0191 when grown under the two cropping systems (Fig S3). Since this carrot genotype lacks genetic resistance, it could theoretically host certain taxa as part of its primary form of defense. Several OTUs were greater in E0191 taproots grown in the organic system (Fig. S3). As described above, *Aspergillus* isolates can benefit carrots by suppressing soft rot and increasing plant growth^112^, and endophytic isolates of *Ophiospharella* can help plants acquire nutrients^118^. While *Fusarium* can act as a pathogen in carrot^104^, many isolates of *Fusarium* can suppress pathogens including pathogenic *Fusarium* species. For example, *Fusarium* endophytes can suppress *F. oxysporum* pathogens in tomato, and *Ustilago maydis* pathogens in maize^120,121^. Two OTUs were significantly greater in E0191 taproots grown in the conventional system (Fig. S3). As described above, *Ceratobasidium* can act as a pathogen in carrot^105^, though endophytic isolates of this genera can also help plants acquire nutrients and fight pathogens^107–109^. *Colleotrichum* has been noted to act as a carrot pathogen^104,122^, indicating that this endophyte could make this genotype more susceptible to other diseases. However, endophytic members of the *Colletotrichum* genus have also been demonstrated to produce bioactive metabolites that work against a number of crop pathogens^123,124^, and help Arabidopsis plants obtain phosphorous^125^.

Finally, the one carrot genotype that did differ in yield between the two management systems in this study (E0252) (Table 3), was also the one genotype that showed no difference in endophyte communities between the management systems. This indicates that other factors, such as greater availability of soil phosphorous, or lower pH between the two systems (Table 2), might have been responsible for the greater productivity of this genotype in the conventional system (Table 3). These results also indicate that this particular genotype could be more discriminative in comparison to other genotypes, with respect to permitting colonization of different endophytes s present in field soil, providing further support for genetic controls on endophyte mycobiomes.

## Conclusions

Carrot taproots host a diverse assortment of fungal endophytes that appear to be part of a core mycobiome unique to carrot. Nevertheless, crop management practices and genotype play a smaller, yet significant role in shaping these communities indicating that it might someday be possible to leverage these communities to enhance crop performance. Our study is only based on one crop season, so it is possible that these communities could change over time, although it was noted that most fungal endophytes in carrot taproots were consistent across years^9^ and we expect the same here. Many of the fungi identified in this trial could positively or negatively affect diseases, so difficulties in isolating fungal endophytes must be overcome so researchers can determine their specific functional roles.

## Supplementary information

Supplementary file1

Supplementary file2

Supplementary file3

Supplementary file4

Supplementary file5

Supplementary file6
