# Supplementary material for: Changes in the core endophytic mycobiome of carrot taproots in response to crop management and genotype
Source: Sci Rep. 2020 Aug 13;10:13685. doi: 10.1038/s41598-020-70683-x (PMC7426841; doi:10.1038/s41598-020-70683-x)
Supplement: Supplementary file 1 — Supplementary file1 [file 41598_2020_70683_MOESM1_ESM.docx]

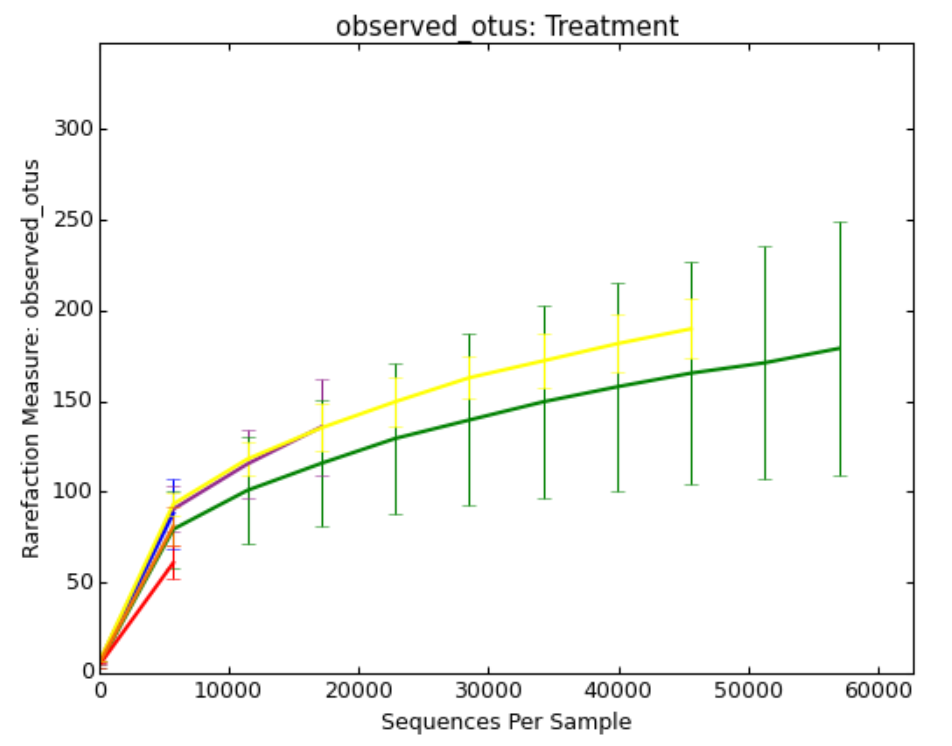


a)


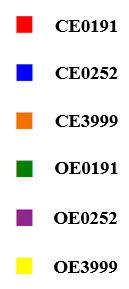


**E0252 and E 3999**

**E0252 and E 3999**

**E0252 and E 3999**

**E0252 and E 3999**


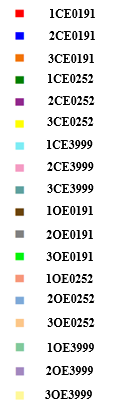

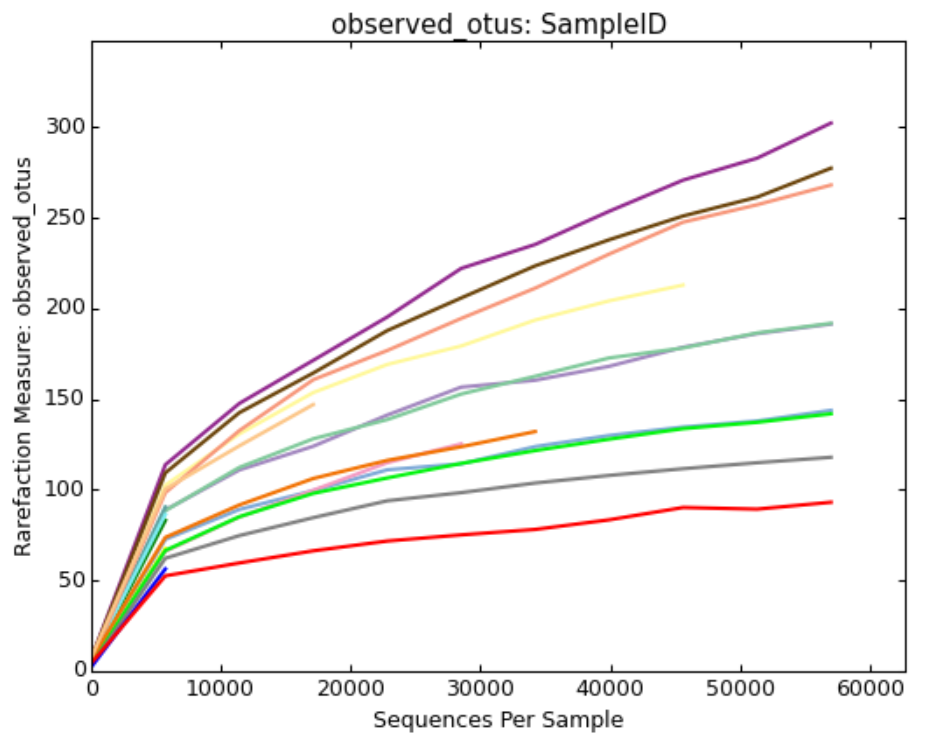


b)

Supplemental Figure 1. Rarefaction curves demonstrated sequencing depth by a) treatment and b) carrot genotype within each treatment.
