# Supplementary material for: Changes in the core endophytic mycobiome of carrot taproots in response to crop management and genotype
Source: Sci Rep. 2020 Aug 13;10:13685. doi: 10.1038/s41598-020-70683-x (PMC7426841; doi:10.1038/s41598-020-70683-x)
Supplement: Supplementary file 2 — Supplementary file2 [file 41598_2020_70683_MOESM2_ESM.docx]

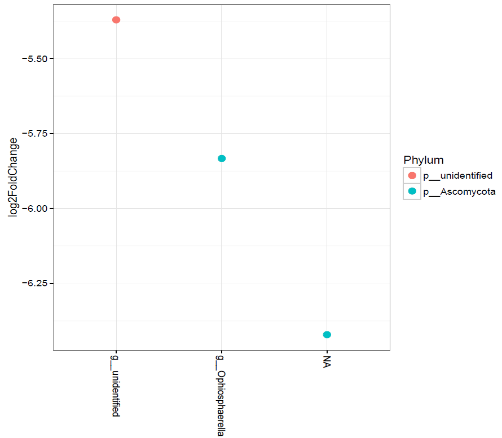

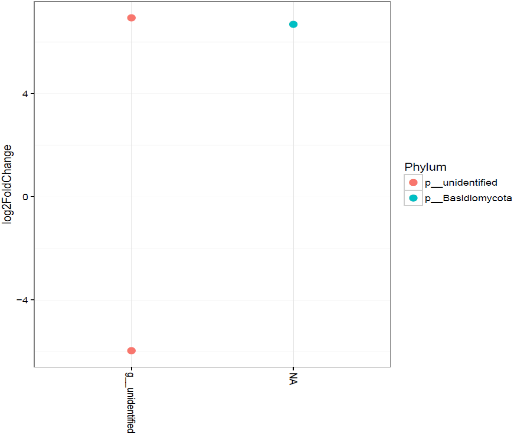

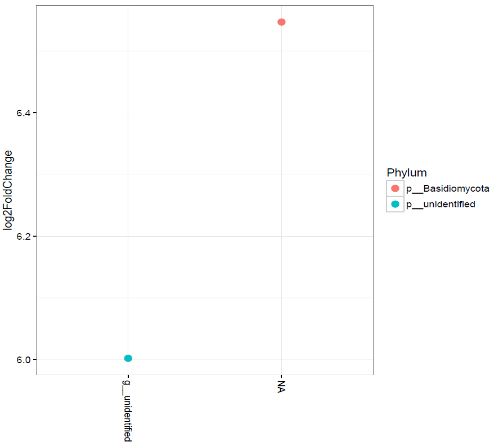

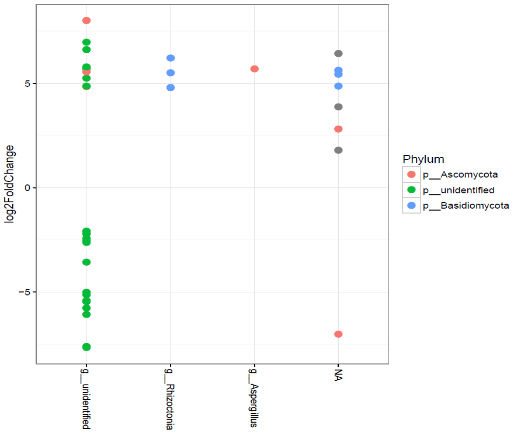

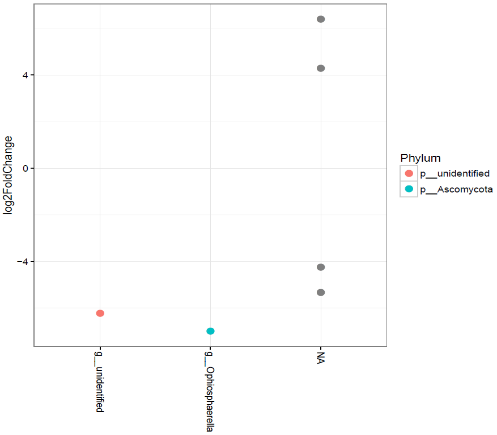

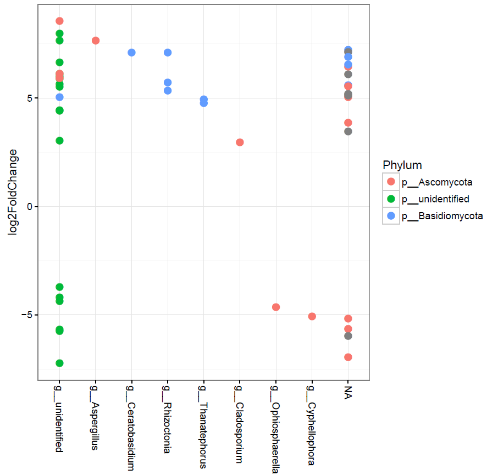

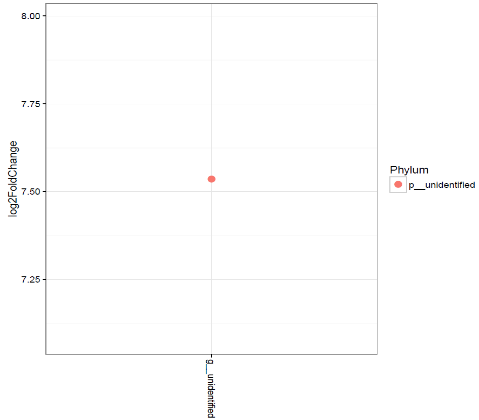

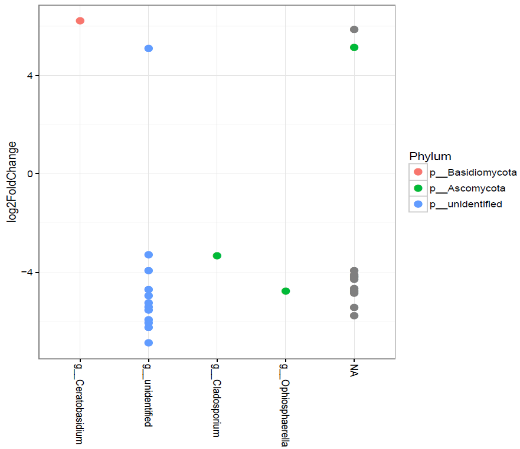

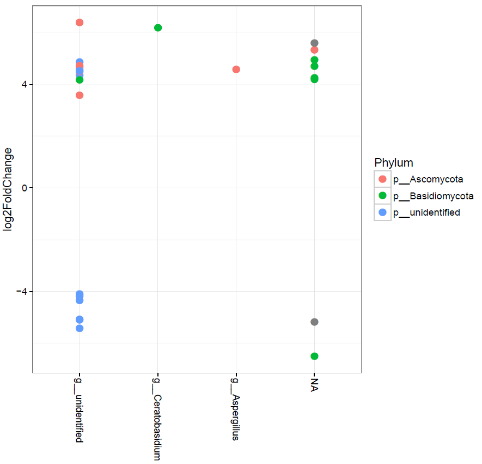


Supplemental Figure 2. Differences in individual fungal endophyte OTU’s between three carrot genotypes across systems or within conventional or organic separately

1. E0252 vs. E3999 (organic)
2. E0252 vs. E3999 (conventional)

g) E0252 vs. E3999 (both systems)

1. E0191 vs. E3999 (organic)
2. E0191 vs. E3999 (conventional)
3. E0191 vs. E3999 (both systems)
4. E0191 vs. E0252 (organic)
5. E0191 vs. E0252 (conventional)
6. E0191 vs. E0252 (both systems)
