# Supplementary material for: Changes in the core endophytic mycobiome of carrot taproots in response to crop management and genotype
Source: Sci Rep. 2020 Aug 13;10:13685. doi: 10.1038/s41598-020-70683-x (PMC7426841; doi:10.1038/s41598-020-70683-x)
Supplement: Supplementary file 3 — Supplementary file3 [file 41598_2020_70683_MOESM3_ESM.docx]

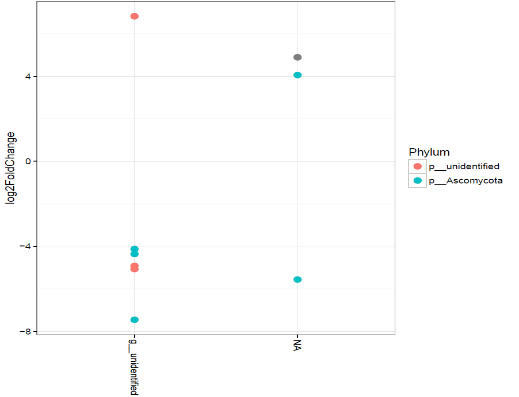

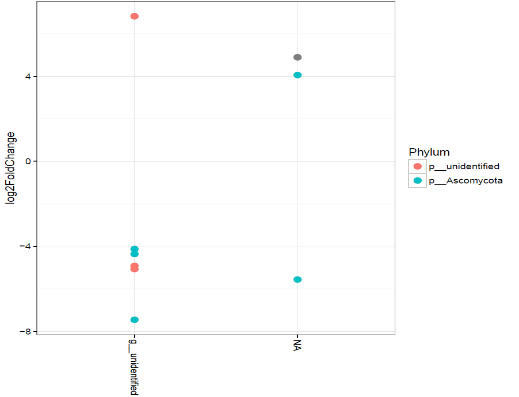

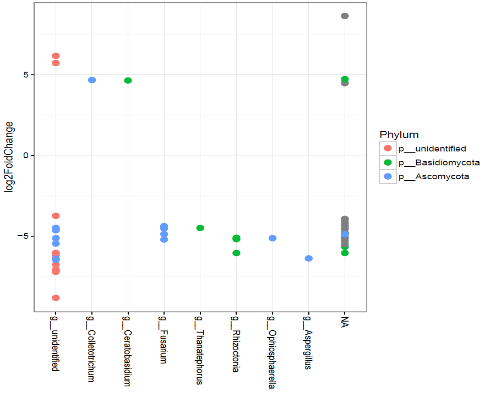

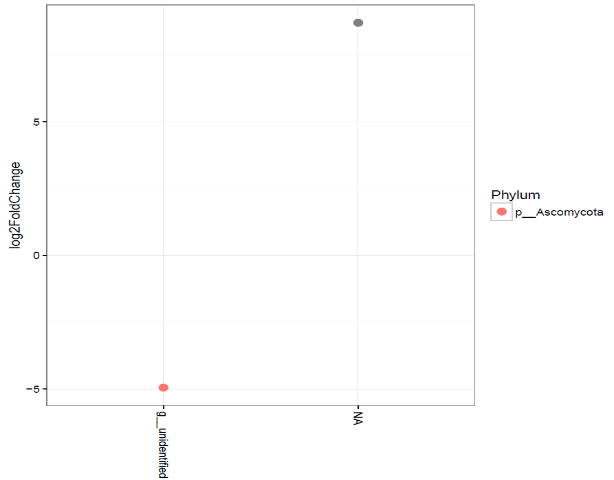


1. E0252 – moderately resistant

Supplemental Figure 3. Differences in individual fungal endophyte OTU’s across organic and conventional among a) all carrot genotypes, and individual carrot genotypes b) E0191 (susceptible), c) E0252 (moderately resistant) and d) E3999 (resistant), representing a range of resistance to root knot nematodes

1. E3999 - resistant
2. E0191 - susceptible
3. All genotypes
